# Multimodal Exploration Offers Novel Insights into the Transcriptomic and Epigenomic Landscape of the Human Submandibular Glands

**DOI:** 10.3390/cells14191561

**Published:** 2025-10-08

**Authors:** Erich Horeth, Theresa Wrynn, Jason M. Osinski, Alexandra Glathar, Jonathan Bard, Mark S. Burke, Saurin Popat, Thom Loree, Michael Nagai, Robert Phillips, Jose Luis Tapia, Jennifer Frustino, Jill M. Kramer, Satrajit Sinha, Rose-Anne Romano

**Affiliations:** 1 Department of Oral Biology, School of Dental Medicine, State University of New York at Buffalo, Buffalo, NY 14214, USA; erichhor@buffalo.edu (E.H.); twrynn@buffalo.edu (T.W.); josinski@buffalo.edu (J.M.O.); jfrustino@ecmc.edu (J.F.); jkramer@buffalo.edu (J.M.K.); 2 Department of Biochemistry, Jacobs School of Medicine and Biomedical Sciences, State University of New York at Buffalo, Buffalo, NY 14203, USA; arglatha@buffalo.edu; 3 Genomics and Bioinformatics Core, State University of New York at Buffalo, Buffalo, NY 14203, USA; jbard@buffalo.edu; 4 Erie County Medical Center, Department of Head & Neck/Plastic & Reconstructive Surgery, Buffalo, NY 14215, USA; mburke@ecmc.edu (M.S.B.); spopat@ecmc.edu (S.P.); tloree@ecmc.edu (T.L.); mnagai@ecmc.edu (M.N.); rphillips1@ecmc.edu (R.P.); 5 Department of Oral Diagnostic Sciences, School of Dental Medicine, State University of New York at Buffalo, Buffalo, NY 14214, USA; jltapia2@buffalo.edu; 6 Erie County Medical Center Division of Oral Oncology & Maxillofacial Prosthetics, Buffalo, NY 14215, USA

**Keywords:** salivary gland, epigenetics, histone modifications, lncRNA, ChIP-seq

## Abstract

The submandibular glands (SMGs), along with the parotid and sublingual glands, generate the majority of saliva and play critical roles in maintaining oral and systemic health. Despite their physiological importance, long-term therapeutic options for salivary gland dysfunction remain limited, highlighting the need for a deeper molecular understanding of SMG biology, particularly in humans. To address this knowledge gap, we have performed transcriptomic- and epigenomic-based analyses and molecular characterization of the human SMG. Our integrated analysis of multiorgan RNA-sequencing datasets has identified an SMG-enriched gene expression signature comprising 289 protein-coding and 75 long non-coding RNA (lncRNA) genes that include both known regulators of salivary gland function and several novel candidates ripe for future exploration. To complement these transcriptomic studies, we have generated chromatin immunoprecipitation sequencing (ChIP-seq) datasets of key histone modifications on human SMGs. Our epigenomic analyses have allowed us to identify genome-wide enhancers and super-enhancers that are likely to drive genes and regulatory pathways that are important in human SMG biology. Finally, comparative analysis with mouse and human SMG and other tissue datasets reveals evolutionary conserved gene and regulatory networks, underscoring fundamental mechanisms of salivary gland biology. Collectively, this study offers a valuable knowledge-based resource that can facilitate targeted research on salivary gland dysfunction in human patients.

## 1. Introduction

The 3 major pairs of salivary glands (SGs) represented by the parotid gland (PG), the sublingual gland (SLG), and the submandibular gland (SMG) serve a crucial role in oral health. The primary task of these exocrine glands is to generate and secrete saliva, which not only aids in food digestion but also facilitates mastication, lubrication of the oral cavity, taste perception, and other myriad processes [1]. The salivary gland comprises various specialized cell types, primarily of epithelial origin, including acinar, ductal, and myoepithelial cells that perform distinct functions and work in concert to produce, modify, and secrete saliva [2,3]. Impaired or diminished SG function in humans, resulting from autoimmune diseases such as Sjögren’s disease, radiation therapy effects in oral cancer patients, or natural aging, leads to hyposalivation. Chronic states of hyposalivation can lead to dysphagia, dental caries, oral infection, and periodontal disease, profoundly affecting the quality of life for patients [4]. To effectively restore the secretory function of the salivary glands, a deeper understanding of their biology, as well as molecular insights into their development, maintenance, and regeneration, remains a research priority of high significance and urgency.

For the past decade, high-throughput next-generation sequencing (NGS) technologies have offered a comprehensive perspective of the SG genomic and epigenomic landscape on a genome-wide scale [5,6]. However, such studies have primarily skewed towards mouse genetic models due to easy accessibility of the tissue. Indeed, bulk and single-cell RNA sequencing (scRNA-seq) as well as epigenomic studies in the mouse SGs from many research groups, including our own, have generated unprecedented close-up views of the cellular complexity of the major SGs and the underpinning gene expression patterns and regulatory networks [5,6,7,8,9,10,11,12,13,14]. However, complementary studies in human SGs have been rather limited, in part due to difficulties in procuring fresh tissue samples that are unafflicted by disease processes. Recent studies have begun to tackle this knowledge gap, as is evident by bulk RNA-seq studies of adult SGs and scRNA-seq datasets that have been generated from human fetal SG and human SMGs and PGs, respectively [15,16,17,18,19]. Notably, similar valuable transcriptomic datasets have also been generated in long-term culture of adult SG organoids, consisting of salivary acinar, ductal, and myoepithelial cells from murine and human major SGs [20]. These studies and subsequent analyses have not only uncovered key developmental and regulatory processes shaping SG-specific functions but also shed light on both broad and cell-type-specific transcriptomic differences among the 3 major SGs as well as between humans and mice.

Recent progress notwithstanding, there remains a pressing need for further molecular investigations of the human SGs, particularly thorough examination of the global epigenomic landscape and its integration with the transcriptome. In this context, our recent work has provided a comprehensive atlas of *cis-regulatory* elements that have allowed for a better understanding of the transcriptional control mechanisms in the mouse SMG and has uncovered a potential role for members of the *FOX* family of transcription factors [12]. Here, we present RNA sequencing (RNA-seq) and chromatin immunoprecipitation sequencing (ChIP-seq)-based studies that first establish a set of human SMG-specific genes and subsequently explore the genome-wide landscape of *cis-regulatory* elements that may regulate these and other key genes that underpin the physiological functions of the SMG. Additionally, we focus on the genome-wide identification and characterization of *cis-regulatory* elements in the human SMG by performing ChIP-seq profiling of histone modifications associated with various classes of gene regulatory elements. These analyses have allowed us to catalog the location and identity of gene regulatory elements, particularly those marking typical enhancers and super-enhancers, on a genomic scale. Notably, our identification of human SMG super-enhancers that are highly enriched with distinct histone modifications and transcription factor binding sites has enabled the discovery of key regulatory regions that drive the expression of genes critical for controlling cell identity. Subsequent clustering of the SMG epigenomic data together with other human tissues, combined with RNA-seq integration, have unearthed interesting molecular players such as transcription factors (TFs) that are enriched and functionally relevant for SMG biology. The resulting epigenomic atlas reported here complements recently generated gene expression datasets, enabling further discovery and investigation of transcriptionally important *cis*- and *trans*-*regulatory* factors that govern SMG cell fate, differentiation, and function.

## 2. Materials and Methods

### 2.1. Collection of Human Submandibular Gland Tissue

SMGs were collected as part of the standard of care for patients undergoing resections for unrelated pathologies at Erie County Medical Center, as previously described [19].

### 2.2. Immunostaining and Imaging

Paraffin-embedded salivary gland sections were processed for immunofluorescence analysis as previously described [9]. Briefly, antigen retrieval was performed with pH 6 sodium citrate buffer, and the sections were blocked with the M.O.M kit (Vector Laboratories, Newark, CA, USA). Primary antibodies used at the indicated dilutions include *SIX1* (1:400, Sigma, St.Louis, MO, USA), TFCP2L1 (1:100, ProteinTech, Rosemont, IL, USA), *FOXC1* (1:50, Cell Signaling Technologies, Danvers, MA, USA), and NKCC1 (1:100, Santa Cruz, Dallas, TX, USA). Sections were stained with TOPRO for nuclear staining, mounted using VECTASHIELD Antifade Mounting Medium (Vector Laboratories), and imaged using an Andor Dragonfly Confocal Microscope with FIJI software (v1.54) [21].

### 2.3. Generation of the Human SMG Gene Signature

The human SMG whole tissue transcriptomic datasets consisting of 21 samples and the scRNA-seq-based results presented in this paper were collected from published data [19]. The processing and analysis pipeline was performed as previously described [10]. To minimize the potential differences and experimental biases in the collection of human samples and to mitigate the inherent batch effects in the processing of RNA-seq datasets, we selected human tissue and organ samples of both male and female sexes and from a wide age range. In addition, the RNA-seq datasets from the 10 tissues and organs were obtained from both the Atlas of Normal Tissue Expression (ANTE) and Encyclopedia of DNA Elements (ENCODE) projects. They consisted of adrenal gland (11 samples), esophagus (11 samples), liver (10 samples), lung (18 samples), mammary gland (6 samples), ovary (10 samples), pancreas (12 samples), skin (10 samples), stomach (8 samples), and thyroid (12 samples) [22,23]. The raw FASTQ files from ANTE and ENCODE databases were downloaded via the SRA-toolkit and processed in the same manner as the human SMG bulk RNA-seq datasets as described above. To examine the relative expression level of genes based on the global transcriptome data and to identify human SMG-specific or -enriched genes, an adjusted *p*-value of < 0.1 (based on the Benjamini–Hochberg method) and a log2FC threshold of +/− 1 were used as a cutoff using DESeq2. The resulting genes were reported as the SMG-specific gene signature.

### 2.4. Salivary Gland Sample Preparation for ChIP-Seq Analysis

Single-cell suspensions were generated from excised submandibular glands by mechanical and enzymatic digestion. The glands were minced with scissors and were incubated in 0.25% trypsin in DMEM/F12 supplemented with 0.5 mg/mL collagenase II (Gibco, Waltham, MA, USA) and 0.5mg/mL dispase II (Roche, Boston, MA, USA). Rocking was performed at 125 rpm for 40 min at 37 °C. The suspension was centrifuged at 400× *g* for 10 min, and the supernatant was discarded. The tissue was resuspended with 0.25% trypsin in DMEM/F12 supplemented with 0.5ml collagenase II (Gibco), 0.5mg/mL dispase II (Roche), and 10 units of DNase I (Zymo Research, Irvine, CA, USA). The tissue suspension was further digested by rocking at 125 rpm for 20 min. Trypsin and collagenase II were inactivated with cold 10% FBS in DMEM/F12. Cell suspension was filtered through a 40 μm nylon filter and was resuspended in 20 mL of DMEM/F12 media for crosslinking by 1% Formaldehyde.

### 2.5. Histone ChIP-Seq

ChIP-seq experiments were performed as previously described [12]. Briefly, the crosslinked SMG single-cell suspensions generated from human tissues were sonicated with a Bioruptor to obtain sheared chromatin to an approximate length of 200–400 bp using a low-SDS shearing kit for histones (Diagenode, Denville, NJ, USA). ChIP experiments were performed using the indirect ChIP protocol (Auto-Histone kit using protein G beads: C01010023) implemented on the IP-Star Compact Automated system. For histone marks, ChIPs were performed using ∼2 μg each of H3K4Me1, H3K4Me2, H3K4Me3, and H3K27Ac antibodies (Diagenode). After cross-linking reversal and proteinase K treatment, the immunoprecipitated and input DNAs were purified using the Qiagen MinElute kit (Qiagen, Germantown, MD, USA), and libraries were prepared using the ThruPLEX DNA-seq kit (Takara Bio, San Jose, CA, USA) from Rubicon Genomics. Library concentration was determined, and the library profile was analyzed on an Agilent 2100 Bioanalyzer (Agilent, Santa Clara, CA, USA). ChIP DNA and input controls were then subjected to single-end next-gen sequencing on an Illumina HiSeq 2500 (Illumina, San Diego, CA, USA). The ChIP-seq data generated for this study has been deposited in the Gene Expression Omnibus (GSE273871).

### 2.6. Histone ChIP-Seq Alignment and Peak Calling

Quality control of raw sequencing reads was performed using FASTQC and subsequently mapped to the Homo sapiens genome (hg38) using Bowtie2 (v2.3.4.1). Resulting SAM files were converted to BAM files and sorted using Samtools (v1.3). Peaks were subsequently called with MACS2 (v2.1.0) using input controls. All ChIP-seq experiments underwent correlation analysis using deepTools (v3.5.0). Samples were then subsequently merged by histone mark type for the remaining downstream analyses. MACS2 peak caller (v2.1.0) was used to identify regions of enriched histone modification signal relative to input DNA control using the following parameters: -*p* 0.01 --nomodel -e 150. ChIPseeker (v1.28.3) was used to annotate the SMG histone modification ChIP-seq peaks to the nearest genomic segment of the hg38 genome assembly. Metaplots showing read distribution proximal to known transcriptional start sites (TSS) in the hg38 genome were generated via deepTools (v3.5.0).

### 2.7. Clustering of Cis-Regulatory Elements

Nucleosome-free regions (NFRs) were defined for the H3K27Ac dataset using the Homer analysis suite (v4.11.1). Clustering of the NFRs utilizing the specific enrichment state of all four histone marks was performed using Fluff (v3.0.3). *De novo* motif enrichment analyses were performed for each cluster using the MEME-ChIP and Tomtom tools available as part of the MEME suite (v5.4.1). Genes proximal to the NFRs for each cluster were identified using GREAT (v4.0.4) using the default settings [24]. Gene set enrichment analyses were performed using DAVID (v2022.q2) and the KEGG database.

### 2.8. Identification of Super-Enhancers in the Human SMG

Using the MACS2-identified H3K27Ac peaks, the ROSE algorithm was employed using the default parameters along with appropriate input controls [25]. *De novo* motif enrichment analysis was performed using the H3K27Ac NFRs identified that overlapped with identified super-enhancers. Similarly to the cluster motif enrichment analyses, we used the MEME suite (v5.4.1) tools—MEME-ChIP and Tomtom. Super-enhancer proximal genes were determined with GREAT (v4.0.4) using the default settings [24]. To determine if the super-enhancer-proximal genes were significantly enriched compared to genes proximal to typical enhancers and genes not proximal to typical enhancers, we used the log ratio normalized counts generated via DESeq2 and considered the mean expression across the 21 human SMG samples that comprised our human SMG gene signature [15,19]. A gene set enrichment analysis was performed on the super-enhancer proximal genes using DAVID (v2022.q2) and the KEGG database. This super-enhancer exploration was repeated with the H3K4Me2 peaks in place of the H3K27Ac peaks.

### 2.9. Identification of Differential Super-Enhancer Loci Across the SMG and Other Human Tissues

The R-package, DiffBind, was used to define the super-enhancers unique to SMG versus a panel of other tissues available through the ENCODE project. ROSE analysis was performed on H3K27ac from each tissue, and the resulting super-enhancer generated was used as input for DiffBind analysis as previously described [12].

## 3. Results

### 3.1. Generation of a Submandibular Gland-Specific Gene Signature

**Figure 1:**
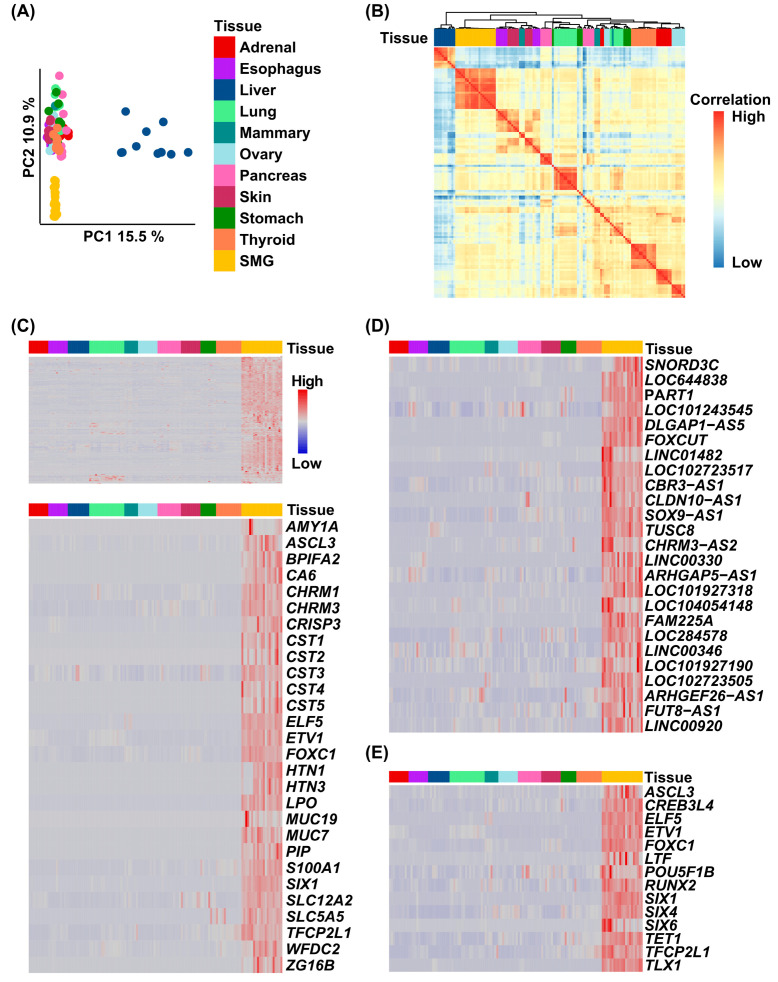
Generation of a tissue-specific human SMG gene signature. (**A**) Principal component analysis (PCA) coordinates from 11 human organs and tissues. (**B**) Correlation plot demonstrating that the human SMGs cluster closely with the esophageal, mammary, and skin. (**C**) Heatmap of the gene expression values selected from the tissue-specific SMG signature across 11 adult human tissues (**upper panel**). The lower panel demonstrates a hierarchical cluster analysis of genes identified in the SMG signature, which have been shown to play a role in salivary gland biology. (**D**) Heatmap of the enriched top 25 lncRNA-coding genes identified in the gene signature. (**E**) Heatmap of the enriched transcription factors identified in the gene signature.

To establish a human SMG-specific gene expression signature, we first compiled a comprehensive set of bulk RNA-seq data, consisting of both newly generated samples from our own study and publicly available datasets generated by Saitou et al. [15,19]. Together, these represented 21 adult human SMG tissue samples, including 13 from males and 8 from females. We then compared these human SMG RNA-seq datasets to those from ten diverse human tissues and organs obtained from the Genotype-Tissue Expression (GTEx) portal and the Atlas of Normal Tissue Expression (ANTE) resource [22]. Utilizing these comprehensive RNA-seq datasets, we first performed a set of initial analyses to examine the relative uniqueness of the global human SMG transcriptome. Principal component analysis (PCA) utilizing 2000 genes with the highest inter-tissue variance revealed that the SMG samples exhibited a gene expression pattern profile sufficiently distinct enough to separate them from all other tissues (Figure 1A). This finding was further supported by hierarchical clustering analysis, which showed the SMG samples clustered together in their own branch of the dendrogram. Interestingly, the SMG exhibited some level of similarities in global transcriptional patterns to esophageal, mammary, and skin samples (Figure 1B).

Next, we employed DESeq2 to perform a differential gene expression analysis with the goal of identifying genes specifically enriched in the SMG. This analysis identified 289 protein-coding genes to be enriched in the SMG compared to all other tissues examined (Figure 1C, upper panel, and Table S1). Interestingly, our analysis also revealed 75 long non-coding RNA (lncRNA) genes, with the top 25 most highly expressed lncRNA presented in Figure 1D and detailed in Table S2. Confirming the biological relevance of the SMG-enriched genes from this analysis is the presence of several well-known players in salivary gland biology (Figure 1C, lower panel), including *SLC12A2,* mucin-7 and -19 (*MUC7* and *MUC19*), and salivary amylase (*AMY1A*). Additional SMG-enriched genes include those encoding for acetylcholine muscarinic receptors (*CHRM3* and *CHRM1*) which mediate parasympathetic control of saliva secretion, and *ZG16B*, a gene proposed as a potential biomarker for salivary gland involvement in graft-versus-host disease [17]. Other notable genes of interest include members of specific families such as cystatins (*CST1-5*), which play protective roles in the oral cavity, acting as natural inhibitors of cysteine proteases. We also observed significant enrichment of proline-rich protein genes—*PRH1-2* and *PRB1-4*—located on chromosome 12p13.2, which form a gene cluster encoding proteins that are predominantly secreted in the saliva (Figure 1C and Table S1). To further investigate the list of human SMG-specific protein-coding genes, we decided to focus on the subset of transcription factors (TFs) enriched in the SMG. Our gene signature includes 14 TFs (Figure 1E), among them *ASCL3*, *ELF5*, and *TFCP2L1*, all of which have previously been implicated in salivary gland biology, primarily through genetic studies in mouse models [26,27,28]. We also identified *FOXC1*, a known driver of SG cell fate [29]. Interestingly, our analysis revealed several other prominent TFs that have not been directly implicated in SMG biology, including *POU5F1B* and *CREB3L4*, which represent promising candidates for follow-up studies.

**Figure 2:**
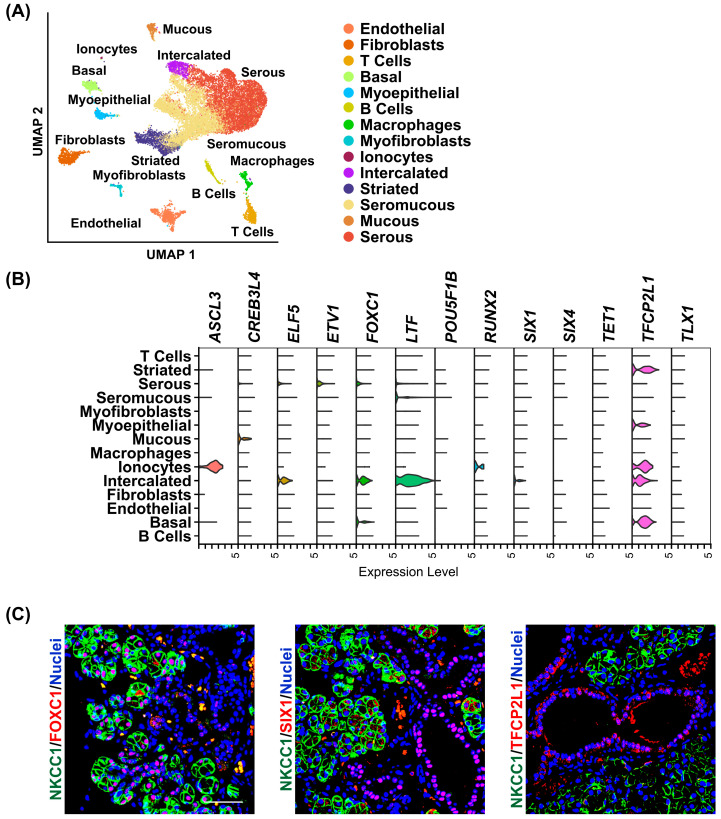
Single-cell RNA-sequencing analysis and expression profile of enriched transcription factors in the human SMG. (**A**) Uniform manifold approximation and projection (UMAP) of the human SMG. Cell cluster identities are also shown in distinct colors. (**B**) Violin plot demonstrating the expression of the enriched transcription factors across the various cell populations as described in panel A above. (**C**) Representative immunofluorescence images of human SMGs stained with a select panel of transcription factors as indicated in red. NKCC1 marks the saliva producing acinar cells (green). Blue = nuclear staining. Scale bar 37.5 μm.

Given the enriched mRNA expression of these TFs, we next sought to examine their localization pattern within the heterogeneous milieu of cell types that are inherent to the human SMG. For this purpose, we leveraged our previously published human SMG scRNA-seq reference dataset, which identified 14 distinct cell populations composed primarily of epithelial cell types and supporting stromal cells such as immune and endothelial cells [19] (Figure 2A). We found that several TFs comprising the SMG gene signature exhibited epithelial-enriched expression, with cell-type specificity ranging from broad epithelial distribution to highly restricted expression in distinct epithelial cell subtypes. For example, we found *ASCL3* and *RUNX2* to be specifically enriched in ionocytes, while *CREB3L4* and *ETV1* were almost exclusively expressed in the mucous and serous cells, respectively (Figure 2B). In contrast, *SIX1*, *FOXC1*, and *ELF5* were predominantly expressed in the intercalated ducts (IDs) [11,12,19,27,30]. These distinct expression patterns are consistent with our previous findings in the SMG and strongly support the possibility that specific TFs work together to coordinate and maintain cell-type specific gene expression patterns in the human SMG [12,27] (Figure 2B). To validate that the transcriptomic profiles correspond to protein expression, we performed immunofluorescence analysis and co-stained human SMG tissue with antibodies against *FOXC1*, *SIX1*, and TFCP2L1 (shown in red), alongside the acinar cell-specific marker NKCC1, shown in green, confirming the cell-type-specific localization of these TFs at the protein level (Figure 2C).

### 3.2. Enriched and Diverse Expression of Important Transcription Factor Families in Human SMGs

Considering that TFs play essential roles in directing tissue organogenesis and homeostasis, it is not surprising that the list of SMG-specific TFs is well represented by members of the erythroblast transformation-specific (ETS), forkhead box (*FOX*), and *sine oculis* (*SIX*) families of proteins. This observation prompted us to examine if these SMG-specific TFs are also among the highest enriched family members in the human SMG. We reasoned the high expression of these TFs would bolster the biological relevance of these genes in salivary gland biology. By considering the mean expression level of each member of a given TF family, we identified the family members most enriched in the salivary gland. Indeed, *ETV1* and *ELF5* were among the most enriched ETS family members, along with *EHF* and *ETV6* (Figure S1A). Interestingly, while our analysis identified *SPDEF* to be highly enriched in the SMG, this gene was not part of the SMG gene signature due to its expression in the stomach (Figure S1A). Similarly, while *FOXC1*, *FOXO3*, and *FOXP1* were among the most highly expressed FOX family members, both *FOXO3* and *FOXP1* did not show SMG-enriched expression in the human [12] (Figure S1B). For the relatively smaller *SIX* family of TFs, we were able to confirm that *SIX1* and *SIX4* are the most enriched members in the human SMG (Figure S1C). Taken together, these findings give credence to the importance of these SMG-enriched TFs and reveal family members that are likely to be important in various biological processes in the SMG.

### 3.3. Generation of a Global Epigenome Map of the Human SMG

**Figure 3:**
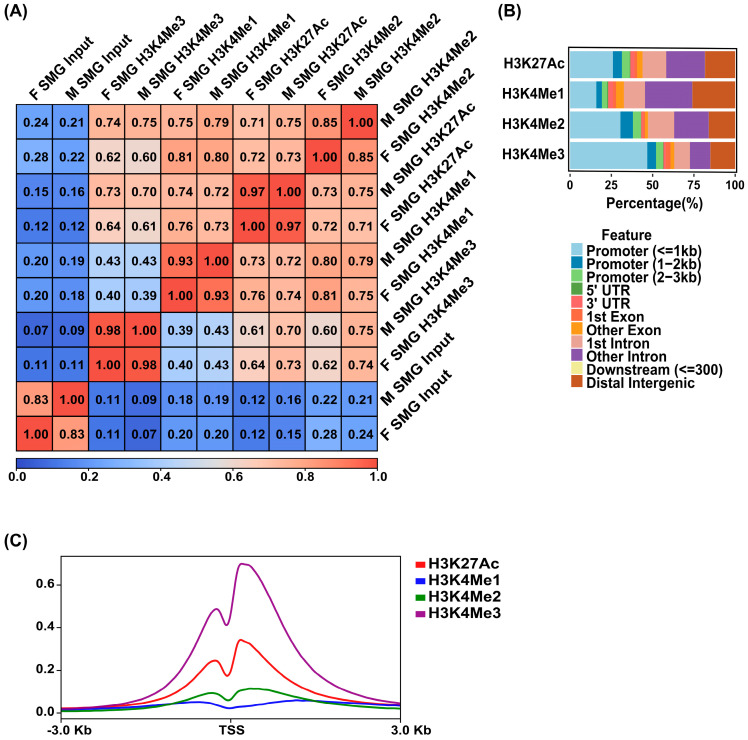
Epigenomic marks in the human SMG. (**A**) Clustered heatmap of the Pearson correlation for the H3K27Ac, H3K4Me1, H3K4Me2, and H3K4Me3 ChIP-seq data based on the normalized read coverage across genome-wide regions. F = Female. M = Male. (**B**) Bar plots showing the percentage distribution patterns and locations of peaks of H3K27Ac, H3K4Me1, H3K4Me2, and H3K4Me3 relative to genes. (**C**) Metaplots of average ChIP-seq density of the 4 histone marks at the TSSs of genes. Enriched H3K4Me3 marks the TSSs of active genes.

While TFs are key drivers of gene regulation, the epigenome, particularly *cis-regulatory* elements such as enhancers and promoters, plays a crucial role in determining both tissue-specific and ubiquitous gene expression programs. To characterize the global epigenomic state of the human SMG, we performed ChIP-seq studies on chromatin isolated from 2 adult human SMG samples (1 male and 1 female). To capture the broad range of regulatory elements, we selected 4 well-established histone modifications: H3K27Ac, H3K4Me2, and H3K4Me1, which mark distinct types of enhancer regions, and H3K4Me3 which is known to be associated with active gene promoters [31,32]. Pairwise Pearson correlation analysis of the male and female human SMG samples showed good concordance between replicates in all 4 histone marks, as evident by the strong correlation coefficient shown across each cell on the clustered heatmap (Figure 3A). Given this high reproducibility, we merged the 2 ChIP-seq replicates for each of the histone mark data using Irreproducible Discovery Rate (IDR). The reproducible peaks across the two replicates ensured more rigor and high-confidence genomic coverage and led to the identification of 55,108 sites associated with H3K27Ac, 159,110 for H3K4Me1, 32,185 for H3K4Me2, and 29,469 for H3K4Me3 peaks. Analysis of the global genome-wide occupancy patterns of the histone marks in the merged datasets showed that H3K27Ac and H3K4Me1 marks are mainly localized within introns or distal intergenic regions (roughly 55% and 70%, respectively). Meanwhile, H3K4Me2 and H3K4Me3 marks were more widely present in promoter-proximal regions (roughly 60% and 70%, respectively) in concordance with established findings (Figure 3B). Focusing more specifically on the transcriptional start site (TSS) of annotated genes, H3K27Ac and H3K4Me3 marks showed robust enrichment in the areas proximal to TSSs, while H3K4Me2 showed a modest enrichment of TSS proximity (Figure 3C). Additionally, the distal enhancer-associated mark, H3K4Me1, showed little TSS proximal enrichment (Figure 3C). Collectively, these results align with the epigenomic findings obtained from our previous studies performed on the mouse SMG [12] and highlight the vast expanse and variety of *cis-regulatory* modules (CRMs) that are operational in the adult human SMG.

### 3.4. SMG-Specific H3K27Ac Peaks Mark Genes Critical to Salivary Gland Function

**Figure 4:**
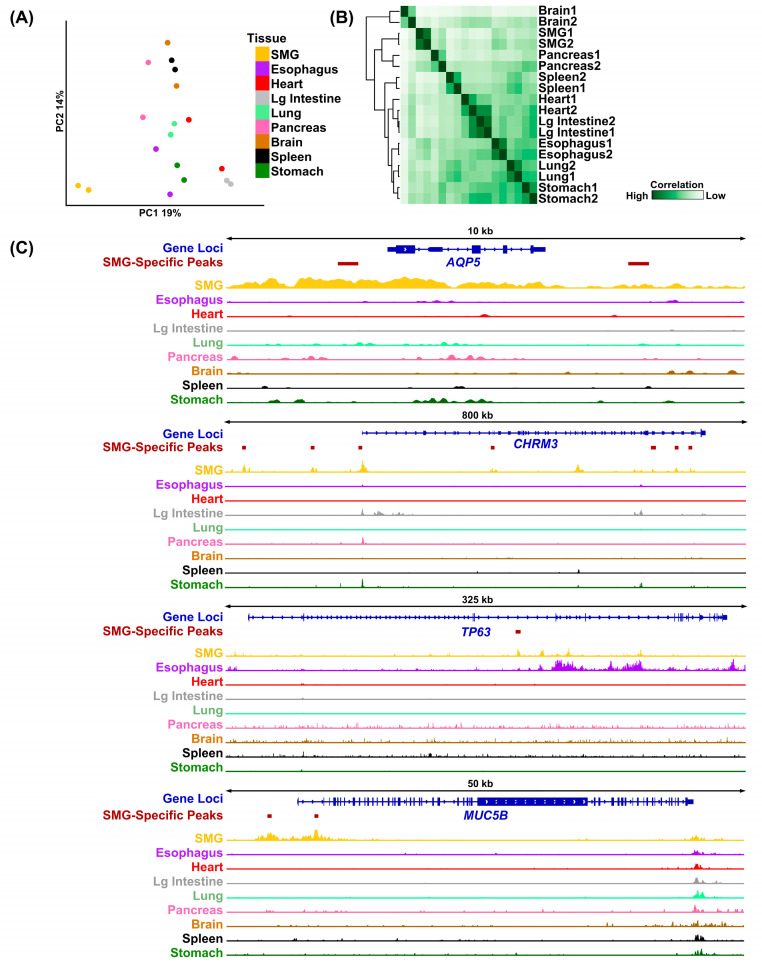
Identification of SMG-specific H3K27Ac peaks. (**A**) PCA coordinates of the human SMG H3K27Ac peaks compared to H3K27Ac peaks of several human tissues for which H3K27Ac ChIP-seq datasets were obtained from the ENCODE project. (**B**) Correlation plot displaying the cross-correlation of H3K27Ac signals across multi-organ human datasets. The SMGs cluster closely with the pancreas, another exocrine gland. (**C**) Visualization of H3K27Ac normalized histone ChIP-seq signals surrounding the genomic loci of *AQP5*, *CHRM3*, *TP63*, and *MUC5B* genes. The locations of the SMG-specific H3K27Ac^hi^ genomic regions in and around the respective gene loci are highlighted. Lg = Large.

Just as specific genes show enriched expression in the SMG, we hypothesized that SMG-specific *cis-regulatory* elements also play a key role in regulating gene expression patterns essential for salivary gland biology. To identify such regulatory regions, we next compared the H3K27Ac-marked peaks in the human SMG to H3K27Ac-marked peaks from multiple human tissues available through the ENCODE project [33]. Utilizing DiffBind, we were able to confirm that the ~50,000 H3K27Ac peaks identified in the SMG represent a unique set of regulatory regions, as shown by PCA (Figure 4A). Hierarchical analysis showed that the SMG samples clustered tightly together and were positioned proximal to the pancreatic tissue, another excretory gland with similar secretory functions (Figure 4B). We next utilized Genomic Regions Enrichment of Annotations Tool (GREAT) [24] to identify genes that are associated with these SMG-specific regulatory regions [34]. This analysis identified 4243 genes associated with SMG-enriched H3K27Ac-marked genomic regions (Table S3). Not surprisingly, several genes that are relevant and feature prominently in SG biology were present in the list, including *AQP5*, *CHRM3*, *TP63*, and *MUC5B* (Figure 4C).

### 3.5. Super-Enhancers in the SMG Likely Regulate Important Genes

**Figure 5:**
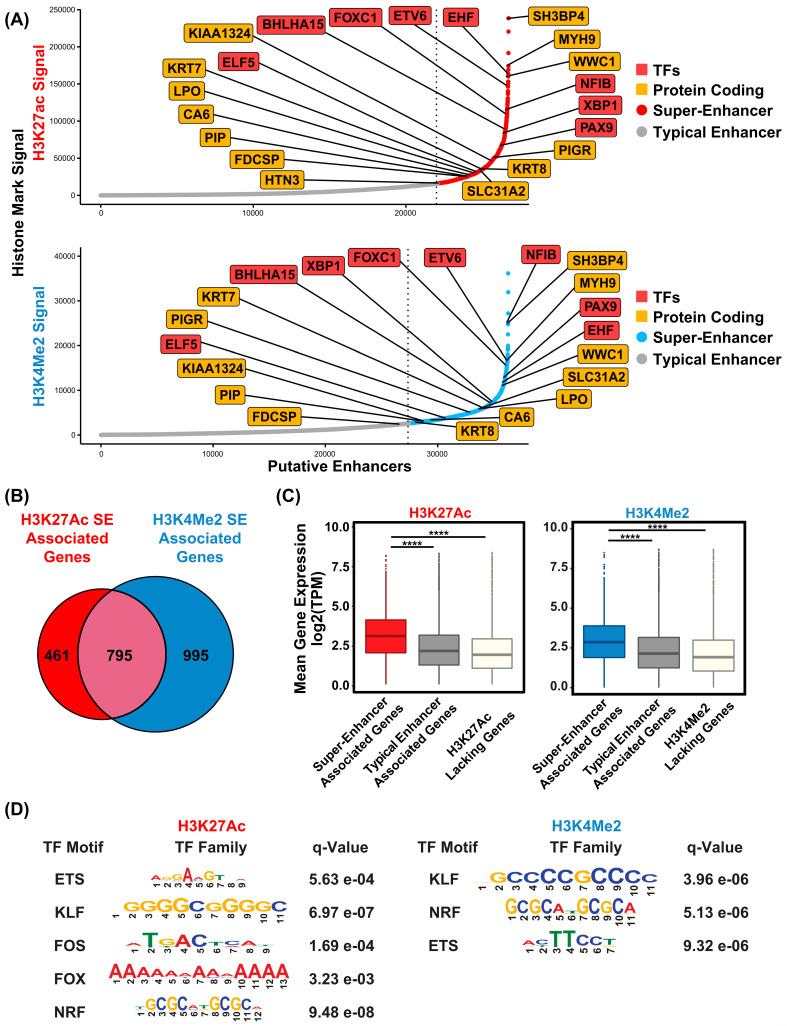
Super-enhancer landscape of the human SMG. (**A**) Hockey stick plot displaying the ranked order of typical enhancers and super-enhancers, as determined by the ROSE algorithm-based analysis of H3K27Ac and H3K4Me2 signals as indicated. Names of a select panel of genes including protein coding (orange squares) and transcription factors (red squares) are shown. SEs are represented by red dots for H3K27Ac signals and blue dots for H3K4Me2 signals, while typical enhancers are denoted with gray dots. (**B**) Venn diagram displaying the overlap of the H3K27Ac and H3K4Me2-marked SEs. (**C**) Boxplot showing the distribution of expression of genes marked by either super-enhancer, typical enhancer, and H3K27Ac lacking genes. **** *p* < 2.2 × 10^−16^ (**D**) Table displaying the top enriched transcription factor motifs found within the SMG super-enhancers.

**Figure 6:**
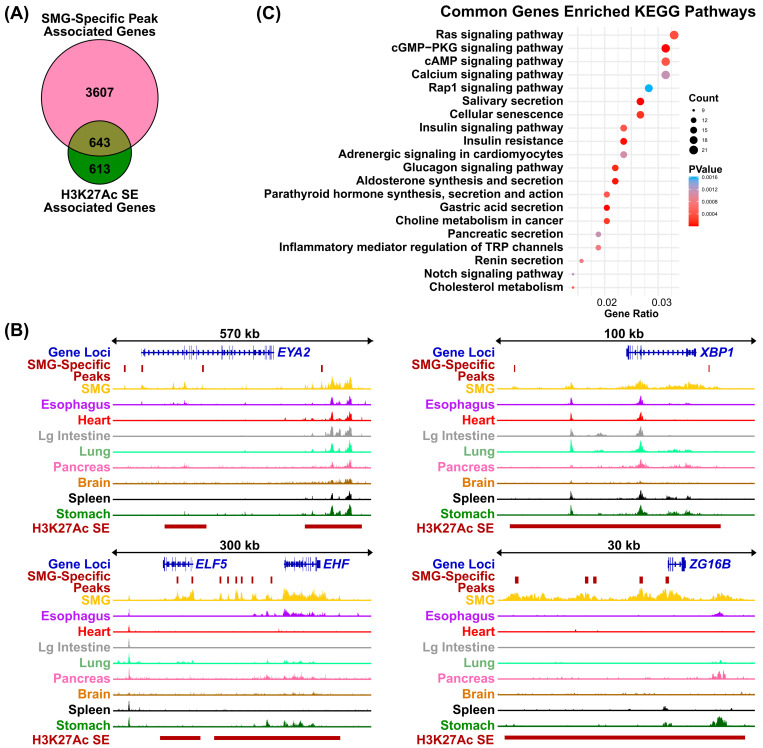
Genomic loci of genes marked by tissue-specific enhancers in the SMG. (**A**) Venn diagram showing SMG-specific, enriched H3K27Ac-marked genomic sites. (**B**) Visualization of normalized H3K27Ac signal across the tissue cohort at the *EYA2*, *XBP1*, *ELF5*, *EHF*, and *ZG16B* genomic loci. The SMG-specific *EYA2*, *XBP1*, *ELF5*, *EHF*, and *ZG16B* super-enhancers, as determined by DiffBind analysis, are highlighted by the solid brown lines. (**C**) KEGG pathways analysis of SMG-specific, enriched H3K27Ac- and super-enhancer-marked genes identified in panel A above.

Compared to typical enhancers, super-enhancers (SEs) are distinguished by their larger size, elevated levels of histone H3 lysine 27 acetylation (H3K27Ac), and increased binding density of transcription factor and coactivator binding [35]. These features enable SEs to act as master regulatory hubs, playing a central role in controlling cell identity, as well as influencing developmental processes and disease states across a wide range of human and mouse cell types. To identify SEs that are present in the human SMG, we applied the Rank Order of Super Enhancer (ROSE) algorithm [25,36] to our SMG H3K27Ac ChIP-seq dataset, using parameters of a stitching distance of 12.5 kb and a ± 2500 bp exclusion zone around the TSS. This analysis identified 1258 genes associated with SEs marked by high levels of H3K27Ac signal (Table S4). As expected, many of the SE-associated genes are known to be highly expressed and/or play important roles in salivary gland development and function, based on prior genetic and biochemical studies [37]. Prominent among them are TFs such as *BHLHA15*, *NFIB*, *FOXC1*, and several members of the ETS transcription factor family (*EHF*, *ELF5*, and *ETV6*) (Figure 5A, upper panel). Interestingly, several highly ranked SE-associated, TFs such as *XBP1* and *PAX9*, are relatively novel, at least in the context of SMG biology, suggesting new avenues of follow-up investigations (Figure 5A, upper panel). Additional SE-associated genes identified include genes spanning diverse biological functions, such as keratins (*KRT7* and *KRT8*), *WWC1*, a key regulator of the Hippo signaling pathway with no previously known role in SMG biology, and *SH3BP4*, a regulator of endocytosis and the highest ranked SE-associated gene identified [38,39]. The discovery of the H3K27Ac-marked super-enhancers in the human SMG prompted us to investigate if the SE-associated genes exhibit a propensity for tissue-specific expression. To address this, we compared the list of 4243 SMG-enriched genes associated with H3K27Ac marked genomic regions with the 1258 SE-associated genes. Remarkably, more than half also overlapped with SMG-specific H3K27Ac peaks, suggesting these SEs contain regulatory regions that are uniquely active in the SMG (Figure 6A, Table S5). In many cases, these SMG-specific enhancers were an integral part of the SE architecture as demonstrated in representative loci such as *EYA2*, *ELF5*, *EHF*, *XBP1*, and *ZG16B* genes (Figure 6B). KEGG pathway analysis of these genes revealed them to be associated with key cellular signaling pathways such as RAS, cAMP, and calcium as well as saliva secretion, further supporting their functional relevance and importance to SMG biology (Figure 6C).

Recent studies have suggested that additional histone modifications, such as H3K4Me2, a mark associated with active transcription, can offer an alternative strategy for identifying SEs [40]. Hence, we applied the ROSE algorithm to the human SMG H3K4Me2 ChIP-seq dataset, which led to the identification of 1644 SE-associated genes (Figure 5A lower panel, Table S6). The H3K4Me2-marked SEs showed similar associations with TFs and other regulatory elements likely to play prominent roles in SMG biology. Importantly, there was robust overlap between SEs identified using H3K4Me2 and those generated from H3K27Ac-based analysis (Figure 5B). Given the prevailing notion that enhancers represent a heterogeneous group with varying levels of strength and functionality [41,42], it is not surprising that SE-associated genes marked by H3K27Ac^hi^ and H3K4Me2^hi^ did not completely overlap. Nevertheless, we found a significant number of SE-associated genes to be shared among both datasets (Figure 5B)—this gene list was considered as a high-confidence dataset and was used for further analyses.

One defining feature of SEs in general is their association with genes that are typically expressed at higher levels compared to genes that are regulated by conventional or typical enhancers. To explore this, we analyzed our previously published RNA-seq datasets to compare expression levels of genes located near SEs versus those near typical enhancers or not associated with any enhancer. Taking into consideration the mean expression values of each gene across all 15 human SMG samples in our dataset, we found that super-enhancer-associated genes common to both the H3K27Ac and H3K4Me2 datasets show significantly higher expression levels than genes linked to typical enhancers or genes lacking enriched H3K27Ac and H3K4Me2 enrichment (Figure 5C). In parallel, we also queried whether genes from the SMG-specific gene signature were associated with SE. Remarkably, a large number of these genes overlapped with SE-associated regions in both the H3K27Ac and H3K4Me2 landscapes (Figure S2), further reaffirming the notion that SE-regulated genes play a crucial role in determining cell and tissue identity by acting as master regulators. To decipher the potential transcriptional networks that may facilitate the expression of these SE-associated genes, we performed motif enrichment analysis to identify the TF motifs that are most highly enriched within the human SMG SE regions (Figure 5D). Not surprisingly, motifs corresponding to the *FOX* and ETS TF families were prominently represented. Since several members of both the *FOX* and ETS families were also identified as SE-proximal genes, we posit that a possible auto-regulatory feedback loop might be in operation in the SMG where key lineage-driving TFs not only regulate important target genes but also direct their own expression through binding to SEs.

### 3.6. Conserved Super-Enhancers Mark Genes Critical to Salivary Gland Function

**Figure 7:**
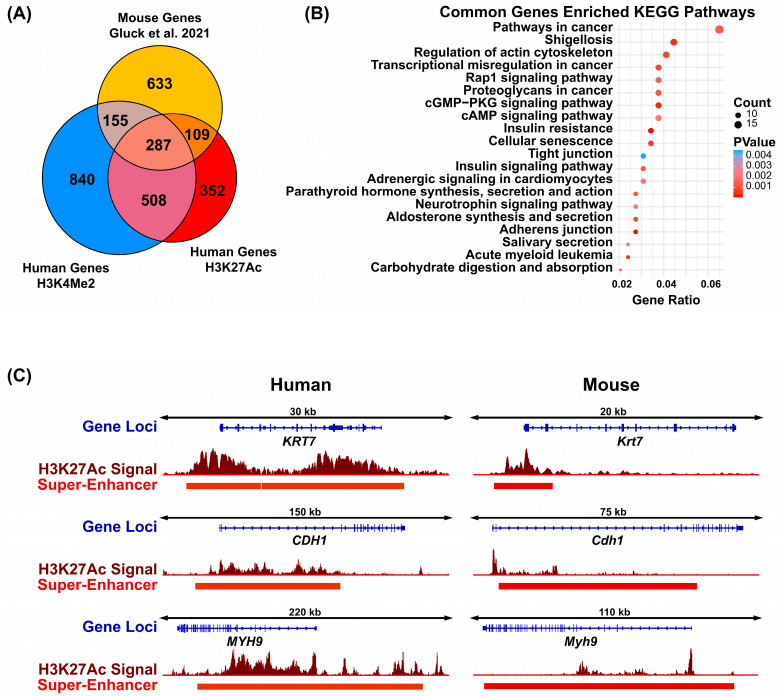
Identification of super-enhancer associated genes conserved between mice and humans. (**A**) Venn diagram displaying overlay of human H3K27Ac SEs, human H3K4Me2 SEs, and mouse high H3K27Ac SEs [12].(**B**) Dot plot of gene set enrichment analysis of the common 287 super-enhancer proximal genes identified in panel (**A**). (**C**) Visualization of super-enhancer signals surrounding the genomic loci of a subset of the 287 super-enhancer associated genes conserved between human and mouse identified in panel (**A**).

We subsequently aimed to evaluate super-enhancers and their associated genes that are conserved between mice and humans. We reasoned that such conserved regions, due to their low rate of evolution—an inherent trait of some functional sequences compared to nonfunctional ones—might reveal *cis-regulatory* regions important for SG biology [43]. Prior studies suggest that while some enhancers are conserved across species, others may have undergone lineage-specific reprogramming as a consequence of human–mouse speciation or divergence. In order to identify super-enhancers that are conserved between mouse and human, we overlaid super-enhancer-proximal genes, that we identified in the human SMG using both the H3K27Ac and H3K4Me2 datasets, with the super-enhancer-proximal genes, that we have previously identified in the mouse SMG based on high H3K27Ac signal [12]. This analysis identified 287 genes that were commonly associated with SEs in all three datasets (Figure 7A and Table S7). Subsequent Gene Set Enrichment Analysis (GSEA) showed that these 287 conserved SE-associated genes are important in salivary gland function, with the pathways represented by this list of genes including the aforementioned Rap1, cGMP-PKG, and cAMP signaling pathways as well as salivary secretion (Figure 7B). To better appreciate the conservation of regulatory elements, we showcase H3K27Ac-marked SE in both human (Figure 7C, left panel) and mouse (Figure 7C, right panel) as evident from ChIP-seq signal pileup plots for three well-known salivary gland epithelial markers: *KRT7*, *CDH1*, and *MYH9*. Taken together, these results reveal the high degree of conservation in super-enhancers between mice and humans, suggesting that genes regulated by these conserved enhancers may represent key regulatory nodes of relevance for future studies in SG biology.

## 4. Discussion

Remarkable progress has been made in deciphering gene expression patterns of various human tissues and organs, largely driven by large-scale initiatives such as the ENCODE and the GTEx consortium projects [44,45]. Despite such efforts, the adult SMG remains one of the few organs for which comprehensive genomic and epigenomic datasets are relatively sparse or entirely lacking. In this study, we present a comprehensive transcriptomic analysis of adult human SMG, alongside the first detailed map of the global *cis-regulatory* elements as defined by key histone modifications. By comparing these newly generated datasets with similar datasets from several other human organs and tissues, we aimed to identify SMG-enriched genes and regulatory elements that are likely to play key roles in the complex multi-cellular processes of this gland. One major motivation for this work is that knowledge generated from such studies will not only advance our understanding of the molecular mechanisms driving the unique transcriptional landscape of the SMG but also lay the groundwork for future studies focused on the regulation of SMG homeostasis, physiology, and disease.

Our comparative and comprehensive analysis of the human SMG transcriptome in relation to other major human organs led to the identification of 289 protein-coding genes and 75 lncRNAs that are enriched in the SMG. Interestingly, the number of genes we identified in our study as SMG-enriched is substantially larger (289 vs. 80) than those reported in a previous publication by Saitou et al. [15]. The differences between our datasets are likely due to differences in sample sizes, analytical methodology, and statistical thresholds used. For instance, our comparison was made exclusively between adult submandibular glands (SMG) and 10 other human tissues/organs, whereas Saitou et al. compared all 3 major adult glands (parotid, sublingual, and submandibular) to 53 tissues and organs. We suspect some of the SMG-enriched genes that are exclusive to our list might have been left out in part due to the stringent nature of the cutoffs that were used in the previously published study. These differences notwithstanding, the 23 genes that are common between our SMG-enriched gene list and those derived by Saitou et al. include *AMY1A*, *MUC19*, and many members of the BPIFA1, CST, and PRB family. Perhaps more strikingly, our analysis also revealed a relatively uncharacterized cohort of lncRNAs that exhibit SMG-specific expression. Given the emerging role of lncRNAs in regulating lineage commitment, cell differentiation, tissue development, and in fine-tuning gene regulatory networks, these lncRNAs represent promising candidates ripe for experimental validation and functional characterization [46].

Our findings reported here address several fundamental questions about the human SMG that have remained largely unanswered. Specifically, we identify and highlight SMG-enriched genes and define the global repertoire of CRMs, including putative enhancers and SEs that regulate their expression. Not surprisingly, we find a large number of genes that are either selectively expressed and/or highly enriched in the SMG and that SMG-specific CRMs likely contribute to their tissue-specific expression. Although there are several well-known candidates in this SMG-specific selected list that have been implicated in SMG biology, many others remain completely new or relatively understudied. Given that some of these genes show similar patterns, such as high expression levels or association with SEs, we posit that these are likely to play important and evolutionarily conserved roles in SG biology and warrant further investigation. In this regard, it is worth pointing out the recent flurry of exciting papers that have honed in on SMG-enriched genes such as *AMY1* and *MUC19*, whose copy number and structural variations, particularly based on archaic genomes of Neanderthals and Denisovans, have offered us new insights into evolutionary selection and human adaptive processes [47,48,49].

Another interesting theme that emerges from our analysis is the prevalence of known gene clusters across the human genome that have been previously shown to be important and relevant for SMG biology. This is best exemplified by a group of structurally related genes encoding the secretory Ca-binding phosphoproteins (SCPPs) gene cluster, spanning ~ 750 kb on chromosome 4q13 [50]. This gene family evolved from a common ancestor gene by tandem duplication events and includes genes encoding salivary proteins, milk caseins, and enamel matrix proteins, which are functionally linked through their role in regulating the calcium phosphate levels in the extracellular environment [51]. The function of the salivary proteins includes protection of tooth enamel by stabilizing saliva supersaturated with calcium salts, as well as antimicrobial and antifungal properties. The expression of the majority of the salivary gland genes within the SCPP gene cluster (e.g., *STATH*, *HTN1*, *HTN3*, *ODAM*, *FDCSP*, and *MUC7*) is mostly restricted to the salivary glands, where they rank among the most highly expressed genes. The extended casein locus is also of particular interest as it harbors at least eight genes expressed in mammary and/or salivary glands. While the five *casein* genes are predominantly expressed in mammary tissue and highly activated during pregnancy, *Fdcsp* is preferentially active in salivary tissue and in immune cells, and *Odam* is expressed in mammary and salivary tissues [52]. The regulatory complexity of this multigene locus with an ancestral super-enhancer active in mammary and salivary tissue suggests shared mechanisms of gene regulation in mammary and salivary glands.

A valuable contribution of our study is the first detailed atlas of potential enhancers and super-enhancers in the human SMG based on several key histone modifications. Together with transcription factors, enhancers are known to play a critical role in orchestrating tissue-specific gene expression patterns during development, homeostasis, and disease. Importantly, a majority of the single nucleotide polymorphisms (SNPs) at disease-associated risk loci identified through genome-wide association studies (GWAS) are located in non-coding regions, and over 90% map to putative enhancer and promoter regions [53,54]. By comparing the global epigenomic landscape of the SMG, particularly H3K27Ac^hi^ regions, across a broad range of human tissues, we provide important insights into the complex transcriptional regulatory mechanisms and the role of putative transcription factors that bind to such enhancers. In particular, we show that there exist many H3K27Ac^hi^ genomic regions that are likely to act as SMG-specific regulatory regions and thus can be harnessed to precisely target and regulate gene expression.

In summary, we have generated and conducted a rigorous and comparative analysis of the gene expression profile and epigenomic state of the human SMG. However, despite the comprehensive nature of our studies, it is worth noting some shortcomings. One obvious caveat is that the enzymatic digestions of the excised salivary gland tissues performed prior to the ChIP experiments could result in inadvertent alterations of the epigenomic landscape. We have tried to mitigate this problem by integrating into our studies transcriptomic data obtained from SMG with limited processing and obviating the technical bias in our datasets by performing replicate experiments and utilizing only the common peaks of the histone mark for downstream analysis. Another limitation of our study is that the current datasets reflect bulk tissue profiles of the adult SMG, without any parsing of the myriad cell types that comprise this complex gland. Although scRNA-seq of adult SMG has been published, a single-cell resolution epigenomic map of the human SMG is still lacking. Future efforts using emerging single-cell and spatial multi-omics technologies that can achieve spatially resolved, genome-wide profiling of both the transcriptome and the epigenome on a relatively unprocessed native tissue sample will pave the way for more detailed molecular mechanisms of how each cell type in the SMG expresses its cognate genes in vivo. A final noteworthy observation is that our studies are limited by the availability of human SMG that skews towards older populations. It will be worthwhile to pursue follow-up investigations into genomic variations in human SMGs across the age spectrum and from different ethnic or racial groups.

## Data Availability

The datasets described in this study have been deposited and can be accessed through accession number GSE273871.

## Supplementary Materials

The following supporting information can be downloaded at: https://www.mdpi.com/article/10.3390/cells14191561/s1, Figure S1: Members of the ETS, *FOX* and *SIX* family of transcription factors are highly enriched in the human SMG, Figure S2: Super-enhancers associated with the genes that comprise the human SMG gene signature, Table S1: Protein coding genes enriched in the SMG, Table S2: LncRNAs enriched in the SMG, Table S3: Genes associated with SMG-enriched H3K27Ac-marked genomic regions, Table S4: Genes associated with SEs marked by high levels of H3K27Ac signal, Table S5: Genes that are associated with both SEs and SMG-specific H3k27Ac marks, Table S6: Human SMG H3K4Me2 SE-associated genes as identified by ROSE algorithm, Table S7: Overlay of common human and mouse SMG SE-associated genes.

## Abbreviations

The following abbreviations are used in this manuscript:

SMGs	Submandibular Gland
lncRNA	Long non-coding RNA
ChIP-seq	Chromatin Immunoprecipitation Sequencing
SGs	Salivary Glands
PG	Parotid Gland
SLG	Sublingual Gland
NGS	Next-generation Sequencing
scRNA-seq	Single-cell RNA-sequencing
TFs	Transcription Factors
ANTE	Atlas of Normal Tissue Expression
PCA	Principal Component Analysis
IDs	Intercalated Ducts
ETS	Erythroblast Transformation-specific
FOX	Forkhead Box
SIX	Sine oculis
IDR	Irreproducible Discovery Rate
TSS	Transcriptional Start Site
CRMs	Cis-regulatory Modules
SE	Super-enhancers
ROSE	Rank Order of Super Enhancer
KRT	Keratin
GSEA	Gene Set Enrichment Analysis
GTEx	Genotype-Tissue Expression
ENCODE	Encyclopedia of DNA Elements
GREAT	Genomic Regions Enrichment of Annotations Tool

## References

[1] Maruyama C.L., Monroe M.M., Hunt J.P., Buchmann L., Baker O.J. (2019). Comparing human and mouse salivary glands: A practice guide for salivary researchers. Oral Dis..

[2] Chibly A.M., Aure M.H., Patel V.N., Hoffman M.P. (2022). Salivary Gland Function, Development and Regeneration. Physiol. Rev..

[3] Amano O., Mizobe K., Bando Y., Sakiyama K. (2012). Anatomy and histology of rodent and human major salivary glands: Overview of the Japan salivary gland society-sponsored workshop. Acta Histochem. Cytochem..

[4] Vissink A., Mitchell J.B., Baum B.J., Limesand K.H., Jensen S.B., Fox P.C., Elting L.S., Langendijk J.A., Coppes R.P., Reyland M.E. (2010). Clinical management of salivary gland hypofunction and xerostomia in head-and-neck cancer patients: Successes and barriers. Int. J. Radiat. Oncol. Biol. Phys..

[5] Gluck C., Min S., Oyelakin A., Smalley K., Sinha S., Romano R.A. (2016). RNA-seq based transcriptomic map reveals new insights into mouse salivary gland development and maturation. BMC Genom..

[6] Gao X., Oei M.S., Ovitt C.E., Sincan M., Melvin J.E. (2018). Transcriptional profiling reveals gland-specific differential expression in the three major salivary glands of the adult mouse. Physiol. Genom..

[7] Song E.C., Min S., Oyelakin A., Smalley K., Bard J.E., Liao L., Xu J., Romano R.A. (2018). Genetic and scRNA-seq Analysis Reveals Distinct Cell Populations that Contribute to Salivary Gland Development and Maintenance. Sci. Rep..

[8] Oyelakin A., Song E.A.C., Min S., Bard J.E., Kann J.V., Horeth E., Smalley K., Kramer J.M., Sinha S., Romano R.A. (2019). Transcriptomic and Single-Cell Analysis of the Murine Parotid Gland. J. Dent. Res..

[9] Min S., Oyelakin A., Gluck C., Bard J.E., Song E.C., Smalley K., Che M., Flores E., Sinha S., Romano R.A. (2020). p63 and Its Target Follistatin Maintain Salivary Gland Stem/Progenitor Cell Function through TGF-beta/Activin Signaling. iScience.

[10] Horeth E., Oyelakin A., Song E.C., Che M., Bard J., Min S., Kiripolsky J., Kramer J.M., Sinha S., Romano R.A. (2021). Transcriptomic and Single-Cell Analysis Reveals Regulatory Networks and Cellular Heterogeneity in Mouse Primary Sjogren’s Syndrome Salivary Glands. Front. Immunol..

[11] Hauser B.R., Aure M.H., Kelly M.C., Hoffman M.P., Chibly A.M., Genomics and Computational Biology Core (2020). Generation of a Single-Cell RNAseq Atlas of Murine Salivary Gland Development. iScience.

[12] Gluck C., Min S., Oyelakin A., Che M., Horeth E., Song E.A.C., Bard J., Lamb N., Sinha S., Romano R.A. (2021). A Global Vista of the Epigenomic State of the Mouse Submandibular Gland. J. Dent. Res..

[13] Sekiguchi R., Martin D., Yamada K.M., Genomics and Computational Biology Core (2020). Single-Cell RNA-seq Identifies Cell Diversity in Embryonic Salivary Glands. J. Dent. Res..

[14] Altrieth A.L., Suarez E., Nelson D.A., Gabunia S., Larsen M. (2023). Single-cell Transcriptomic Analysis of Salivary Gland Endothelial Cells. bioRxiv.

[15] Saitou M., Gaylord E.A., Xu E., May A.J., Neznanova L., Nathan S., Grawe A., Chang J., Ryan W., Ruhl S. (2020). Functional Specialization of Human Salivary Glands and Origins of Proteins Intrinsic to Human Saliva. Cell Rep..

[16] Chu T., Wang Z., Pe’er D., Danko C.G. (2022). Cell type and gene expression deconvolution with BayesPrism enables Bayesian integrative analysis across bulk and single-cell RNA sequencing in oncology. Nat. Cancer.

[17] Costa-da-Silva A.C., Aure M.H., Dodge J., Martin D., Dhamala S., Cho M., Rose J.J., Bassim C.W., Ambatipudi K., Hakim F.T. (2022). Salivary ZG16B expression loss follows exocrine gland dysfunction related to oral chronic graft-versus-host disease. iScience.

[18] Chen M., Lin W., Gan J., Lu W., Wang M., Wang X., Yi J., Zhao Z. (2022). Transcriptomic Mapping of Human Parotid Gland at Single-Cell Resolution. J. Dent. Res..

[19] Horeth E., Bard J., Che M., Wrynn T., Song E.A.C., Marzullo B., Burke M.S., Popat S., Loree T., Zemer J. (2023). High-Resolution Transcriptomic Landscape of the Human Submandibular Gland. J. Dent. Res..

[20] Yoon Y.J., Kim D., Tak K.Y., Hwang S., Kim J., Sim N.S., Cho J.M., Choi D., Ji Y., Hur J.K. (2022). Salivary gland organoid culture maintains distinct glandular properties of murine and human major salivary glands. Nat. Commun..

[21] Schindelin J., Arganda-Carreras I., Frise E., Kaynig V., Longair M., Pietzsch T., Preibisch S., Rueden C., Saalfeld S., Schmid B. (2012). Fiji: An open-source platform for biological-image analysis. Nat. Methods.

[22] Suntsova M., Gaifullin N., Allina D., Reshetun A., Li X., Mendeleeva L., Surin V., Sergeeva A., Spirin P., Prassolov V. (2019). Atlas of RNA sequencing profiles for normal human tissues. Sci. Data.

[23] Consortium E.P. (2012). An integrated encyclopedia of DNA elements in the human genome. Nature.

[24] McLean C.Y., Bristor D., Hiller M., Clarke S.L., Schaar B.T., Lowe C.B., Wenger A.M., Bejerano G. (2010). GREAT improves functional interpretation of cis-regulatory regions. Nat. Biotechnol..

[25] Whyte W.A., Orlando D.A., Hnisz D., Abraham B.J., Lin C.Y., Kagey M.H., Rahl P.B., Lee T.I., Young R.A. (2013). Master transcription factors and mediator establish super-enhancers at key cell identity genes. Cell.

[26] Arany S., Catalan M.A., Roztocil E., Ovitt C.E. (2011). Ascl3 knockout and cell ablation models reveal complexity of salivary gland maintenance and regeneration. Dev. Biol..

[27] Song E.A.C., Smalley K., Oyelakin A., Horeth E., Che M., Wrynn T., Osinski J., Romano R.A., Sinha S. (2023). Genetic Study of Elf5 and Ehf in the Mouse Salivary Gland. J. Dent. Res..

[28] Yamaguchi Y., Yonemura S., Takada S. (2006). Grainyhead-related transcription factor is required for duct maturation in the salivary gland and the kidney of the mouse. Development.

[29] Tanaka J., Ogawa M., Hojo H., Kawashima Y., Mabuchi Y., Hata K., Nakamura S., Yasuhara R., Takamatsu K., Irie T. (2018). Generation of orthotopically functional salivary gland from embryonic stem cells. Nat. Commun..

[30] Rheinheimer B.A., Pasquale M.C., Genomics N.N., Limesand K.H., Hoffman M.P., Chibly A.M., Genomics and Computational Biology Core (2023). Evaluating the transcriptional landscape and cell-cell communication networks in chronically irradiated parotid glands. iScience.

[31] Barral A., Dejardin J. (2023). The chromatin signatures of enhancers and their dynamic regulation. Nucleus.

[32] Preissl S., Gaulton K.J., Ren B. (2023). Characterizing cis-regulatory elements using single-cell epigenomics. Nat. Rev. Genet..

[33] Consortium E.P. (2011). A user’s guide to the encyclopedia of DNA elements (ENCODE). PLoS Biol..

[34] Tanigawa Y., Dyer E.S., Bejerano G. (2022). WhichTF is functionally important in your open chromatin data?. PLoS Comput. Biol..

[35] Hnisz D., Abraham B.J., Lee T.I., Lau A., Saint-Andre V., Sigova A.A., Hoke H.A., Young R.A. (2013). Super-enhancers in the control of cell identity and disease. Cell.

[36] Loven J., Hoke H.A., Lin C.Y., Lau A., Orlando D.A., Vakoc C.R., Bradner J.E., Lee T.I., Young R.A. (2013). Selective inhibition of tumor oncogenes by disruption of super-enhancers. Cell.

[37] Mellas R.E., Kim H., Osinski J., Sadibasic S., Gronostajski R.M., Cho M., Baker O.J. (2015). NFIB regulates embryonic development of submandibular glands. J. Dent. Res..

[38] Zhong Z., Jiao Z., Yu F.X. (2024). The Hippo signaling pathway in development and regeneration. Cell Rep..

[39] Burckhardt C.J., Minna J.D., Danuser G. (2021). SH3BP4 promotes neuropilin-1 and alpha5-integrin endocytosis and is inhibited by Akt. Dev. Cell.

[40] Fox S., Myers J.A., Davidson C., Getman M., Kingsley P.D., Frankiewicz N., Bulger M. (2020). Hyperacetylated chromatin domains mark cell type-specific genes and suggest distinct modes of enhancer function. Nat. Commun..

[41] Narita T., Higashijima Y., Kilic S., Liebner T., Walter J., Choudhary C. (2023). Acetylation of histone H2B marks active enhancers and predicts CBP/p300 target genes. Nat. Genet..

[42] Das N.D., Chang J.C., Hon C.C., Kelly S.T., Ito S., Lizio M., Kaczkowski B., Watanabe H., Katsushima K., Natsume A. (2023). Defining super-enhancers by highly ranked histone H4 multi-acetylation levels identifies transcription factors associated with glioblastoma stem-like properties. BMC Genom..

[43] Frazer K.A., Elnitski L., Church D.M., Dubchak I., Hardison R.C. (2003). Cross-species sequence comparisons: A review of methods and available resources. Genome Res..

[44] Consortium G.T. (2013). The Genotype-Tissue Expression (GTEx) project. Nat. Genet..

[45] Shi M., Mear L., Karlsson M., Alvez M.B., Digre A., Schutten R., Katona B., Vuu J., Lindstrom E., Hikmet F. (2025). A resource for whole-body gene expression map of human tissues based on integration of single cell and bulk transcriptomics. Genome Biol..

[46] Statello L., Guo C.J., Chen L.L., Huarte M. (2021). Gene regulation by long non-coding RNAs and its biological functions. Nat. Rev. Mol. Cell Biol..

[47] Yilmaz F., Karageorgiou C., Kim K., Pajic P., Scheer K., Beck C.R., Torregrossa A.M., Lee C., Gokcumen O., Human Genome Structural Variation Consortium (2024). Reconstruction of the human amylase locus reveals ancient duplications seeding modern-day variation. Science.

[48] Bolognini D., Halgren A., Lou R.N., Raveane A., Rocha J.L., Guarracino A., Soranzo N., Chin C.S., Garrison E., Sudmant P.H. (2024). Recurrent evolution and selection shape structural diversity at the amylase locus. Nature.

[49] Villanea F.A., Peede D., Kaufman E.J., Anorve-Garibay V., Chevy E.T., Villa-Islas V., Witt K.E., Zeloni R., Marnetto D., Moorjani P. (2025). The MUC19 gene: An evolutionary history of recurrent introgression and natural selection. Science.

[50] Pajic P., Landau L., Gokcumen O., Ruhl S. (2025). Saliva protein genes in humans were shaped during primate evolution. Genome Biol. Evol..

[51] Haller F., Bieg M., Will R., Korner C., Weichenhan D., Bott A., Ishaque N., Lutsik P., Moskalev E.A., Mueller S.K. (2019). Enhancer hijacking activates oncogenic transcription factor NR4A3 in acinic cell carcinomas of the salivary glands. Nat. Commun..

[52] Lee H.K., Willi M., Liu C., Hennighausen L. (2023). Cell-specific and shared regulatory elements control a multigene locus active in mammary and salivary glands. Nat. Commun..

[53] Corradin O., Scacheri P.C. (2014). Enhancer variants: Evaluating functions in common disease. Genome Med..

[54] Gasperini M., Tome J.M., Shendure J. (2020). Towards a comprehensive catalogue of validated and target-linked human enhancers. Nat. Rev. Genet..

