# Supplementary material for: Multimodal Exploration Offers Novel Insights into the Transcriptomic and Epigenomic Landscape of the Human Submandibular Glands
Source: Cells. 2025 Oct 8;14(19):1561. doi: 10.3390/cells14191561 (PMC12524052; doi:10.3390/cells14191561)
Supplement: Supplementary file 1 [file cells-14-01561-s001.zip › cells-3869680-supplementary-author proof done.v1.pdf]

*cells*

*Article*

# Multimodal Exploration Offers Novel Insights into the Transcriptomic and Epigenomic Landscape of the Human Submandibular Glands

**Erich Horeth [1,†], Theresa Wrynn [1,†], Jason M. Osinski [1], Alexandra Glathar [2], Jonathan Bard [3], Mark S. Burke [4], Saurin Popat [4], Thom Loree [4], Michael Nagai [4], Robert Phillips [4], Jose Luis Tapia [5], Jennifer Frustino [1,6], Jill M. Kramer [1], Satrajit Sinha [2,*], and Rose-Anne Romano [1,2,*]**

 https://doi.org/10.3390/cells14191561

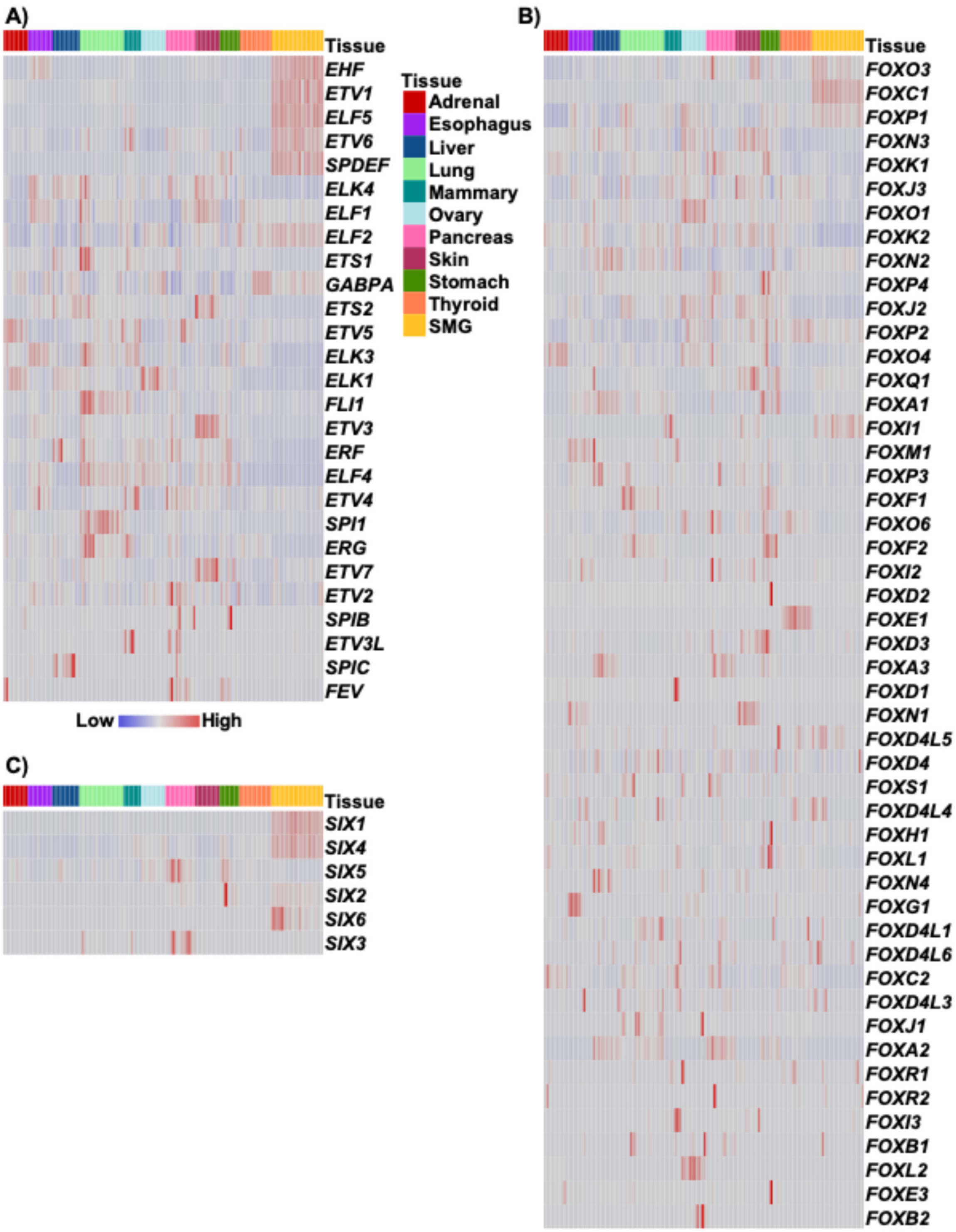


**Supplemental Figure S1.** Members of the ETS, FOX and SIX family of transcription factors are highly enriched in the human SMG. Heatmap showing the expression of (**A**) ETS, (**B**) FOX, and (**C**) SIX family of transcription factors in human SMG across a panel of human organs and tissues.

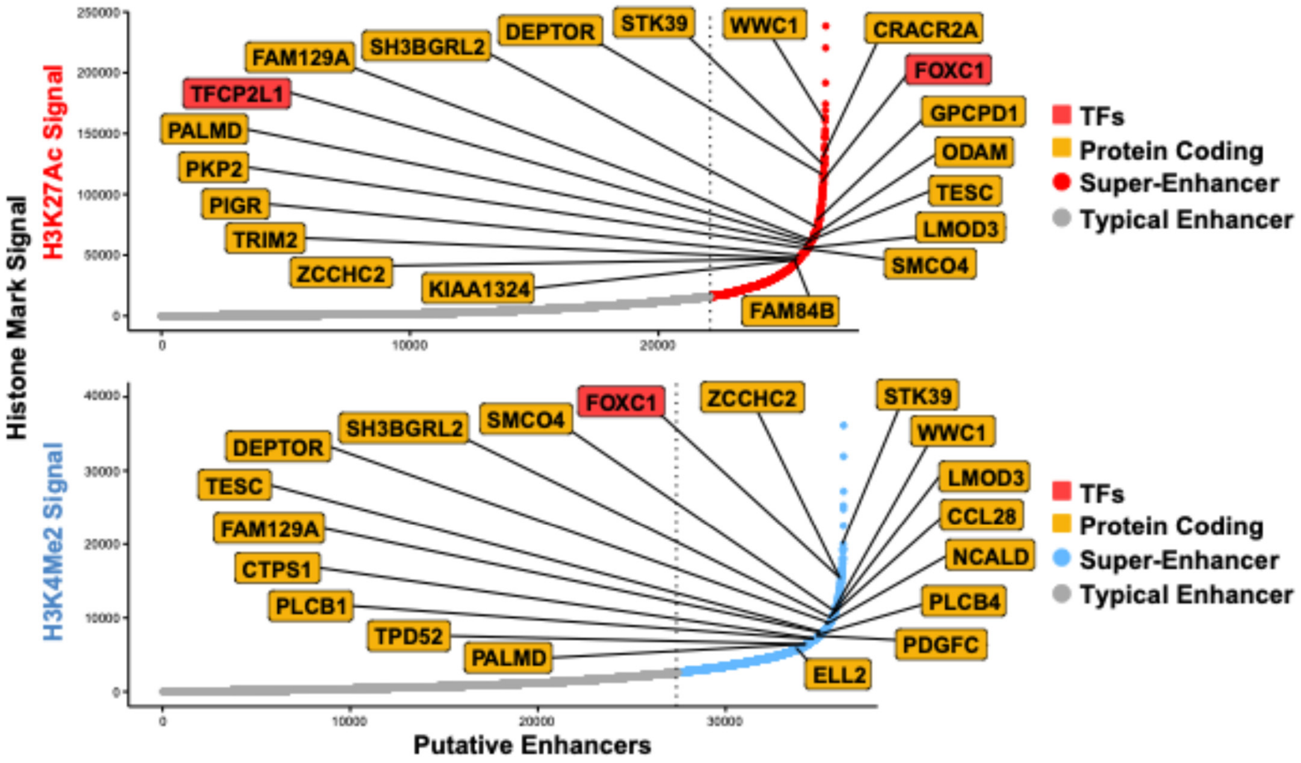


**Supplemental Figure S2.** Super-enhancers associated with the genes that comprise the human SMG gene signature. Hockey stick plot displaying the ranked order of typical enhancers and super-enhancers as determined by the ROSE algorithm for H3K27Ac and H3K4Me2 signals as indicated, for genes that are part of the human SMG gene signature. Names of a select panel of genes including protein coding (orange squares) and transcription factors (red squares) are shown. SE's are represented by red dots for H3K27Ac signals and blue dots for H3K4Me2 signals while typical enhancers are denoted with grey dots.
